# Exploring the contribution of exposure heterogeneity to the cessation of the 2014 Ebola epidemic

**DOI:** 10.1371/journal.pone.0210638

**Published:** 2019-02-01

**Authors:** Florian Uekermann, Lone Simonsen, Kim Sneppen

**Affiliations:** 1 Niels Bohr Institute, University of Copenhagen, Copenhagen, Denmark; 2 Department of Science and Enviroment, Roskilde University, Roskilde, Denmark; Mercy Hospital, SIERRA LEONE

## Abstract

The unexpected early cessation of the recent West Africa Ebola outbreak demonstrated shortcomings of popular forecasting approaches and has not been fully understood yet. A popular hypothesis is that public health interventions mitigated the spread, such as ETUs and safe burials. We investigate whether risk heterogeneity within the population could serve as an alternative explanation. We introduce a model for spread in heterogeneous host population that is particularly well suited for early predictions due to its simplicity and ease of application. Furthermore, we explore the conditions under which the observed epidemic trajectory can be explained without taking into account the effect of public health interventions. While the obtained fits closely match the total case count time series, closer inspection of sub-population results made us conclude that risk heterogeneity is unlikely to fully explain the early cessation of Ebola; other factors such as behavioral changes and other interventions likely played a major role. More accurate predictions in a future scenario require models that allow for early sub-exponential growth, as well as access to additional data on patient occupation (risk level) and location, to allow identify local phenomena that influence spreading behavior.

## Introduction

The devastating 2014 outbreak of Ebola in West Africa was unexpected, as previous outbreaks have been far smaller. A “perfect storm” of a broken health care system, increased mobility, recent unrest and low public trust were cited to explain the unprecedented scale of the epidemics [1].

Based on the assumption of prolonged exponential growth of the epidemic, exceedingly dire predictions were disseminated by both scientific press [2] and news media outlets [3]. However, the spread of the epidemic slowed down quicker than expected and eventually ceased, even though the vast majority of people were still susceptible to the virus. Similarly, early modeling efforts to predict the likely trajectory in the autumn of 2014 greatly overestimated the size and duration of the outbreak.

A modeling analysis of different scenarios after September 2014 predicted a case count of 170 000 for Montserrado county (Liberia) in a worst case scenario and 40 000 cases in a best case with significantly increased mitigation efforts [4]. Based on data until the end of August 2014, another study forecasted a total of 550 000 reported cases in Liberia and Sierra Leone until January 20, 2015 [5]. A worst case estimate from August 2014 projected 80 000 cases or more during the remainder of the year [6]. Even projections based on a model considering a decaying reproduction number suggested that the epidemics could reach a size of more than 140 000 cases, based on the case counts until August 2014 [7]. However, all of these forecasts significantly exceed the final case count of less than 30 000 cases across the three most heavily affected countries Guinea, Sierra Leone and Liberia [8], which corresponds to less than 0.2% of the population.

A systematic review of modeling studies published during the epidemic shows that the examples above are not isolated cases [9]. The discrepancy between prediction and data is largely rooted in an unexpectedly fast decline of the reproduction number. As basic S(E)IR models assume constant and homogeneous spread, epidemics are expected to end only after a substantial part of the population have become immune. In the West Africa epidemic however, the fraction of the suceptible population removed from the dynamics due to Ebola infection remained negligible. This suggests that other mechanisms restricted the long-term growth of the epidemics, leading to dramatically overestimated final case counts in predictions based on traditional S(E)IR models.

A variety of mitigation mechanisms have been proposed as explanations [10], but not all mechanisms have been studied with respect to the Ebola epidemic in West Africa. These explanations can be separated into two broad classes: (1) The observed slowdown is caused by deliberate mitigation strategies, such as individual efforts to avoid risk, as well as population-wide efforts, such as the establishment of ETUs and improved testing, prevention and treatment. (2) The observed slowdown is caused by intrinsic properties of the host-virus epidemiology, which will always lead to a rapid cessation of the outbreak. Multiple studies have investigated how different kinds interventions of class 1 may affect the transmission of the virus [4, 11, 12]. This study supplements these efforts by investigating the impact of a mechanism of class 2 on transmission. In particular we propose heterogeneity in the population with respect to the risk of contracting Ebola could be an overlooked (intrinsic) factor. This is motivated by a similar situation in the 1980s, when modelers initially greatly overestimated the trajectory of the global HIV/AIDS epidemic, based on it’s initial rapid growth. It has subsequently been demonstrated that population heterogeneity is key to explaining the observed slowdown in the spread of HIV [13, 14]. Furthermore, other studies support a generally quicker decline of the reproduction number, in case of population heterogeneity [15–19].

Given that both HIV and Ebola transmission require close contact with infected individuals, it seems fitting to consider that differences in behavior due to occupation and behavior may influence an individuals risk of infection. In fact, surveillance data suggests that health care workers as a group were at 100-fold higher risk than the general population in the 2014 outbreak [20].

Here we explore a simple model that takes heterogeneous contact rates into account, by introducing a small population with high risk of contracting the virus. We show that a good fit for the declining effective reproduction number *R*_*t*_ and the time-series of infections can be obtained with this model. Using agent-based simulations we also explore the models sensitivity to the stochastic nature of disease spreading dynamics.

The heterogeneous spreading model introduced in this paper is unique in its simplicity and small number of parameters. Its application requires only estimates of the size and the basic reproduction number of the high risk population as additional parameters, while removing the need to estimate the total population size. Furthermore the effective reproduction number can be calculated in one step from the cumulative case count at any point in time. The model is intended as an easily applicable tool in the early stages of future epidemics like the Ebola epidemic in West Africa, where risk heterogeneity is likely to play a role.

## Model

**Fig 1:**
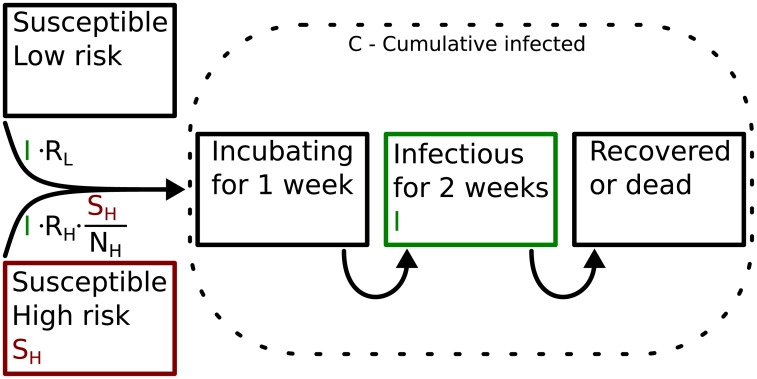
Schematic representation of the SEIR model with a small highly susceptible subpopulation. The transition rates from susceptible to incubating are expressed in number of infections per 2 weeks (the infectious time of an infected host).

We consider a scenario with two subpopulations: A large population with low risk of contracting Ebola, and a small high risk population, possibly representing health care workers or other caretakers with higher exposure to the pathogen. We use a SEIR model with a fully mixed population (E represents the incubation period). The sizes of the high- and low-risk populations are denoted by *N*_*H*_ and *N*_*L*_. Their respective basic reproduction numbers are *R*_*H*_ and *R*_*L*_. See Fig 1 for a model schematic.

This distinction is only considered relevant for the risk of infection of healthy individuals. No distinction is made between infected individuals from either population. Consequently any primary case will result in secondary cases in both populations according to their respective basic reproduction number (*R*_*H*_ and *R*_*L*_). Thus the basic reproduction number for the population as a whole is *R*_0_ = *R*_*H*_ + *R*_*L*_.

In this paper we consider a scenario where the low risk population is much larger than the high risk population (*N*_*L*_ >> *N*_*H*_). Furthermore, the low-risk population has a basic reproduction number significantly smaller than one (*R*_*L*_ < 1). These constraints are motivated by the desire to minimize the number of parameters of the model, to aid application of the model even if little data beyond the time-series of infection counts is known.

### Agent based model

The assumption of a small high risk population and a small low risk reproduction number *R*_*L*_, generally results in epidemics where removal of susceptible individuals in the low-risk population is negligible. As a result the size of the low risk population *N*_*L*_ is not relevant to the spreading dynamics. This allows us to simplify the effective reproduction number *R*(*t*), by only accounting for the depletion of susceptible hosts in the high risk population:

$$\begin{array}{cc} R(t) & =R_{L}+R_{H}\cdot \frac{S_{H}\left(t\right)}{N_{H}}\\ S_{H}\left(0\right) & =N_{H}\\ \Rightarrow R(0) & =R_{L}+R_{H}=R_{0} \end{array},$$ (1)

where *S*_*H*_(*t*) represents the number of people in the high risk population that are susceptible (have never been infected) at time *t*.

### Simplified differential equation model

Assuming a large number of infections we can describe the resulting dynamics in terms of differential equations (see Materials and methods for details). This leads to an expression for the effective reproduction number *R*(*t*), that only depends on the cumulative number of all infections *C*(*t*) at time *t* and the size of the subpopulation *N*_*H*_. With this approach we achieve the simplicity of an SIR differential equation model in the otherwise much more complex case of two interacting subpopulations:

$$\begin{array}{c} R\left(t\right)=R_{L}+R_{L}\cdot W(\frac{R_{H}}{R_{L}}\cdot e^{\frac{R_{H}}{R_{L}}(1-\frac{C(t)}{N_{H}})}) \end{array}$$ (2)

*W* represents the “product logarithm” (see Materials and methods).

This is the model equation we will fit to the data.

## Results

Only the results for Liberia are shown in the main text. See supplement S1 Additional Figures for Sierra Leone, Guinea and additional figures.

**Fig 2:**
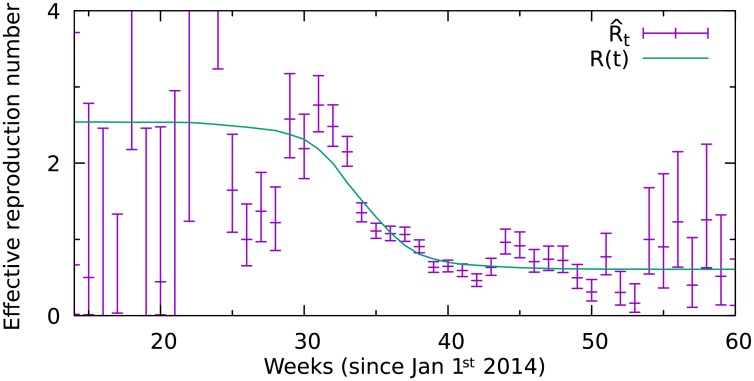
Weekly estimates of the effective reproduction number ${\hat{R}}_{t}$ in Liberia (purple crosses). Fitted *R*(*t*) (green line) with *R*_*H*_ = 2.0, *R*_*L*_ = 0.6 and *N*_*H*_ = 2500 (see Eq 2).

Fig 2 shows weekly estimates ${\hat{R}}_{t}$ of the reproduction number since 1st of January 2014 (Eq 3). Up to week 33 the reproduction number is substantially greater than 1 and does not show a substantial decline. However by week 38 it declines to less than 1, entering the sub-critical regime where it remains without changing recognizably after week 40.

Note that the uncertainty of individual weekly estimates in the beginning and end of the epidemics is naturally large, because the number of infected is small and subject to comparatively large fluctuations. However, in combination we can use these individual estimates to fit *R*_*t*_ as described by Eq 3 (green line).

From this fit we obtain not only a smooth estimate *R*_*t*_, but also estimates for the reproduction number in the high- and low risk population (*R*_*s*_, *R*_*n*_) and the size of the high risk population (*N*_*s*_), which are shown in Table 1.

**Table 1:** Parameters and results of best *R*(*t*)-fit (see Eq 2 and green line in Fig 2).

	Sierra Leone	Liberia	Guinea
Observation			
Total cases	12*k*	6*k*	4*k*
Model			
Predicted cases (*C*_∞_)	12k	6*k*	3.5*k*
*R*_0_ = *R*_*H*_ + *R*_*L*_	1.5	2.5	1.3
*R*_*H*_ (high risk)	1.3	2.0	1.2
*R*_*L*_ (low-risk)	0.1	0.6	0.1
high risk population (*N*_*H*_)	16k	2.5k	6.2k
Cases in high risk population	10.5k	2.5k	3.4k

### Parameter sensitivity

**Fig 3:**
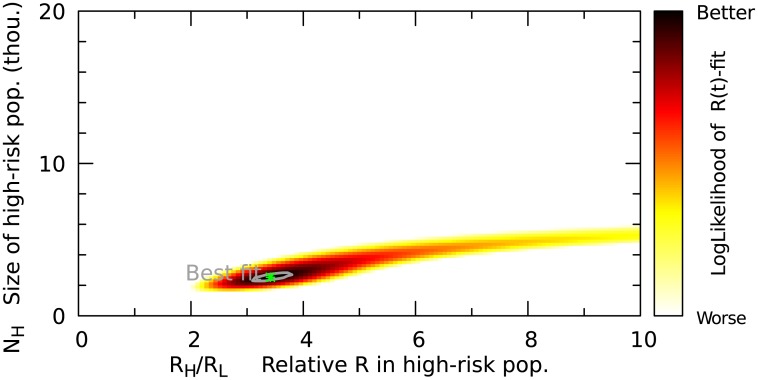
Quality of the fit shown in Fig 2 for different parameter choices. The grey line marks the 95% confidence interval.

Fig 3 shows the likelihood surface of the *R*_*t*_-fit for a range of value combinations of *R*_*s*_, *R*_*n*_ and *N*_*s*_. The maximum likelihood values (green star) within the shown parameter range were used for the *R*_*t*_-curve in Fig 2. However, there is a range of parameter combinations that result in good fits of the weekly reproductive numbers shown in Fig 2. Especially the value of *R*_*s*_/*R*_*n*_ allows for variation. This factor describes how much higher the high risk populations contribution (*R*_*s*_) to the basic reproduction number is, compared to the low-risk populations contribution (*R*_*n*_). For example *R*_*s*_/*R*_*n*_ = 2 would imply that at the beginning of the epidemics each primary case generates on average twice as many secondary cases in the high risk population as in the low-risk population.

It is important to note that *R*_*s*_ and *R*_*n*_ already account for different subpopulation sizes, such that the basic reproduction number is simply *R*_0_ = *R*_*s*_ + *R*_*n*_. To compare the risk of infection for individuals we have to take into account that the high risk population is much smaller than the low-risk population. The individual risk of infection in the high risk population compared to the low-risk population is higher than *R*_*s*_/*R*_*n*_ by a factor *N*/*N*_*s*_ (*N*: total population).

The corresponding *R*_0_ for each parameter combination is shown in Fig F in S1 Additional Figures. However, for any reasonable fit *R*_0_ doesn’t vary much because it must agree with the *R*_*t*_ values from early case counts (see Fig 2). For this reason we show the fit-quality over the two remaining parameters *N*_*s*_ and *R*_*s*_/*R*_*n*_.

**Fig 4:**
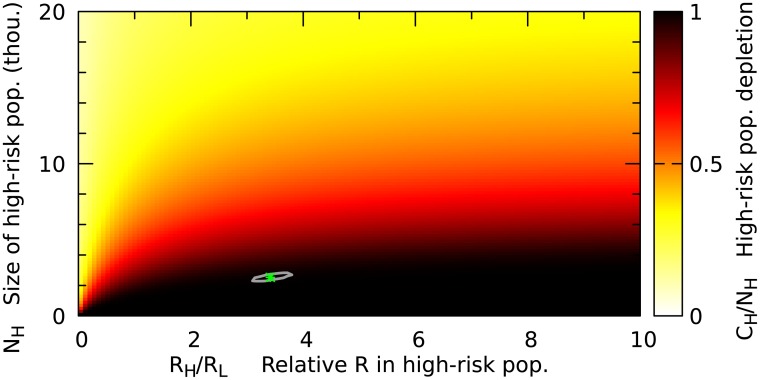
Expected final fraction of infected high risk population for different parameter choices. The matching *R*_0_ for is shown in Fig F in S1 Additional Figures.

After obtaining the range of reasonable parameters we can make predictions for the final fraction of the subpopulation that will be infected (Fig 4) and the final epidemics size (Fig E in S1 Additional Figures).

Health-care workers (HCWs) are an obvious candidate as members of the high risk population. According to Kilmarx et al. [20] approximately 5% of the infected in Sierra Leone were HCWs, which indicates about 100 fold higher risk of infection for HCWs. However, even in the light of these numbers the number of high risk cases predicted by our model seems unrealistically high. However, there are multiple possible explanations for this: 1) The high risk population may not include all HCW, but only those working in particularly dangerous conditions. 2) The model only takes symptomatic cases into account, where the victim is also infectious. 3) The high risk population may not primarily consist of HCW, but other people who are at higher risk due to their particular environment or behavior.

Of particular interest for scenario 2) is that 27% are estimated to be asymptomatic [21]. The model can be adapted to account for this by scaling the total size of the high risk population accordingly. All other parameters and predictions remain unchanged under the assumption that asymptomatic infections are typically not transmitted.

### Agent based simulations

**Fig 5:**
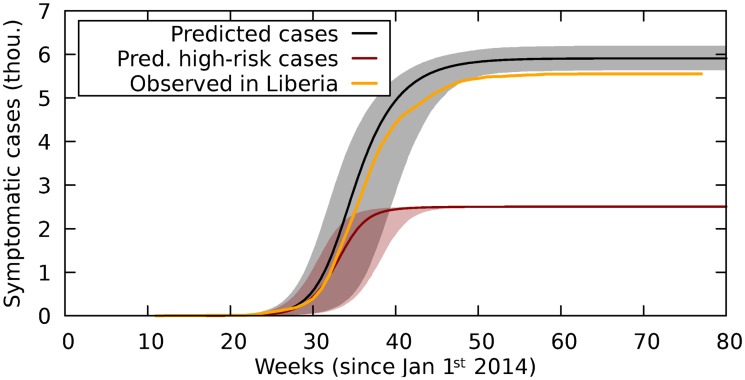
Observed case count and results of the agent based simulations using the fitted parameter set (see green star in Fig 3 and fit in Fig 2). Orange line: Observed case count in Liberia. Black line: Mean total case count. Red line: Mean case count in the high risk population. Grey and red areas: Prediction interval (95%) for case counts (95% of our simulations produce case counts within this area).

To further investigate the validity and uncertainty of our predictions we have to take the stochastic nature of real world infection spreading into account. Using an agent based implementation of our agent based model (see Materials and methods for details) we can simulate this stochastic dynamics. Fig 5 shows the averaged simulation results for the total case number, the number of cases in the high risk population and the corresponding prediction intervals. The simulations use the fitted parameter set we use in Fig 2.

For both Liberia (Fig 5) and Sierra Leone (see S1 Additional Figures) the number of observed infections tends to be slightly lower than the prediction. In Liberia this trend leads to a final epidemic size that is slightly smaller than the prediction interval. This could be a due to stochastic nature of the process, an artifact of the data, or an indication that relief efforts and behavioral adaptation contributed to the decreased spreading, which is not accounted for in this model.

### Other countries

All figures in the main text only show our results for Liberia. The corresponding figures for Sierra Leone and Guinea are shown in the supplementary material S1 Additional Figures, along with additional figures for all countries. Compared to Liberia, for Sierra Leone and Guinea better fits are achieved with a larger relative difference in susceptibility between high risk and low-risk population ($\frac{R_{s}}{R_{n}}$). To allow easy visual comparison between the three countries we constrained the explored parameter ranges to $\frac{R_{s}}{R_{n}}\in \left[0,10\right]$ and *N*_*s*_ ∈ [0, 20 000]. Even though the best likelihood is achieved for larger $\frac{R_{s}}{R_{n}}$, we obtain a good fit of *R*_*t*_ and matching case number predictions for Sierra Leone.

In the case of Guinea the estimates for *R*_*t*_ show large fluctuations with at least 2 cycles of alternating high and low effective reproduction number. This likely indicates unreliable data or external factors, such as a re-ignition of the epidemics from the outside. Due to the small total number of cases in Guinea (< 4000), of which many are located at the borders to Sierra Leone and Liberia, we expect that the dynamics in Guinea is influenced by cases across it’s borders.

Our model predictions for Guinea match the reported case counts roughly. Allowing for a larger $\frac{R_{s}}{R_{n}}$ factor would increase the similarity further. However, both explanations we considered for the fluctuations in *R*_*t*_ (unreliable data or external influence) make the case counts from Guinea unsuitable to evaluate our model.

## Discussion

Given the assumption of a heterogeneous population with a small high risk population, we formulate a minimal 3 parameter SEIR-model for the spreading dynamics of Ebola in West Africa in 2014. Our model reproduces the early exponential growth, with *R*_0_ > 2 in some places [8], as well as the cessation of the epidemic.

However, the best fits to the case count data require a much higher risk of infection (> 1000 fold) in the high risk population and attribute nearly half of all cases to the high risk population. One such subpopulation that has been identified are health care workers (HCW), with an approximately 100-fold elevated risk [20] accounting for less than 10% of all cases.

Both a broader definition of the high risk population, including other caretakers and people engaging in risky behavior, or further differentiation of subpopulations may reconcile this discrepancy. Alternatively, additional factors could be considered, such as behavioral changes on an individual level or mitigating interventions as discussed in other modeling studies of this [11] and previous epidemics [22]. In particular we favor the idea that individuals are increasingly cautious after experiencing Ebola in their local community. This hypothesis of learned caution is supported by the observation that the local spreading on a district level often stopped after about 100 days [23]. This is long enough for population heterogeneity to play a role, but also strongly suggests a local transition from members of high risk to lower-risk subpopulations after the epidemics reached a local community. These local patterns are easily missed when looking at aggregated national data, since the spread to new regions obscures the local cessation, causing apparent sustained exponential growth.

While it seems unlikely, that our model includes all important factors that an accurate model of this epidemic would require, it showcases a simple extension of traditional S(E)IR models, that greatly improves the prediction of the effective reproduction number and the projected case counts. Our results, the observation of increased risk of infection in the HCW population [20], suggest that the homogeneous population approach, which works well in the case of respiratory viruses may not be sufficient for viruses that require close contact for transmission to occur (Ebola, HIV).

Our study as well as previous work on HIV model overestimation highlight that early predictions regarding the spread of a pathogen that requires close contact would greatly benefit from more detailed observed data. Early availability of data regarding patient occupation and geographical location would allow modelers to identify risk heterogeneity and adjust their predictions accordingly. Going forward, pandemic threats will be evaluated in context of conclusions drawn from outbreaks such as the 2014 Ebola epidemics. Our minimal and easily applicable model may be useful for early predictions in cases where intrinsic features of the contact network make naive *R*_0_ estimates unsuitable for long term predictions. Also, a look at the local Ebola epidemics suggest behavioral changes may have a mitigating effect in affected areas, which deserves more study. Ultimately, a robust understanding of population patterns of risk heterogeneity and existence of behavioral changes in exposed populations will add to models ability to produce more accurate forecasts during a future epidemic emergency.

## Materials and methods

### Estimating R from data

We use the weekly incidence (counts) of confirmed and suspected symptomatic cases in the patient database data published by the WHO [24]. The data consists of a cumulative weekly case count. The incubation period of 1 week, followed by a 2 week infectious period (accounting for symptomatic cases and unburied corpses). This results in an average serial interval of 2 weeks (time between primary to secondary infection). No distinction is made between fatal and non-fatal courses of disease. Assuming that the infection rate is constant within one week, these spreading parameters allow us to estimate a weekly effective reproduction number ${\hat{R}}_{t}$:

$$\begin{array}{c} {\hat{R}}_{t}=\frac{d_{t+1}}{\frac{d_{t}}{4}+\frac{d_{t-1}}{2}+\frac{d_{t-2}}{4}}=\frac{\text{new}\;\text{inf.}\;\text{in}\;\text{week}\;\text{t}}{\text{sympt.}\;\text{during}\;\text{week}\;\text{t}}, \end{array}$$ (3)

where *d*_*t*_ represents the number of reported new symptomatic cases in week *t*. The evolution of ${\hat{R}}_{t}$ over time is shown in Fig 2.

### Agent based simulations

Our agent based simulations are a stochastic implementation of the model described at the beginning of this section (Fig 1, Eq 1). We start with 1 infectious agent on day 0. A random number of agents from each population is infected per timestep (Δ*t* = 2.4h). The probabilities for the number of infections are distributed according to the infection rates (Fig 1, Eq 1). Infected agents become infectious after an incubation period of 1 week and are removed from the simulation 2 weeks later (death or recovery).

### Derivation of *R*(*t*)

Given large enough infection numbers, the number of new infections in the subpopulation per infections in the whole population can be described by:

$$\begin{array}{cc} \frac{dC_{H}\left(t\right)}{dC(t)} & =\frac{R_{H}\cdot \frac{S_{H}\left(t\right)}{N_{H}}}{R_{H}\cdot \frac{S_{H}\left(t\right)}{N_{H}}+R_{L}}\\ & =\frac{R_{H}\cdot \left(1-\frac{C_{H}\left(t\right)}{N_{H}}\right)}{R_{H}\cdot \left(1-\frac{C_{H}\left(t\right)}{N_{H}}\right)+R_{L}} \end{array}$$

This leads to the following function for the cumulative number of infections in the subpopulation:

$$\begin{array}{c} C_{H}\left(t\right)=N_{H}-\frac{R_{L}}{R_{H}}N_{H}\cdot W(\frac{R_{H}}{R_{L}}e^{\frac{R_{H}}{R_{L}}(1-\frac{C(t)}{N_{H}})}) \end{array}$$

Finally, substituting *S*_*H*_(*t*) with *N*_*H*_ − *C*_*H*_(*t*) in Eq 1 yields Eq 2:

$$\begin{array}{cc} R(t) & =R_{L}+R_{H}\cdot \frac{N_{H}-C_{H}\left(t\right)}{N_{H}}\\ R(t) & =R_{L}+R_{L}\cdot W(\frac{R_{H}}{R_{L}}\cdot e^{\frac{R_{H}}{R_{L}}(1-\frac{C(t)}{N_{H}})}) \end{array}$$

Note: *W* represents the “Product logarithm” or “Lambert W function”, which is defined by:

$$\begin{array}{c} z=W\left(z\right)\cdot e^{W(z)} \end{array}$$

## Supporting information

S1 Additional Figures Figures for Sierra Leone, Guinea and additional figures for Liberia. (PDF)
