# Supplementary material for: Exploring the contribution of exposure heterogeneity to the cessation of the 2014 Ebola epidemic
Source: PLoS One. 2019 Feb 1;14(2):e0210638. doi: 10.1371/journal.pone.0210638 (PMC6358083; doi:10.1371/journal.pone.0210638)
Supplement: S1 Additional Figures — (PDF) [file pone.0210638.s001.pdf]

S1 - Additional figures

January 20, 2019

$$R(t) = R_L + R_L \cdot W\left(\frac{R_H}{R_L} \cdot \mathrm{e}^{\frac{R_H}{R_L}\left(1 - \frac{C(t)}{N_H}\right)}\right) \tag{1}$$

W represents the "product logarithm"

Liberia

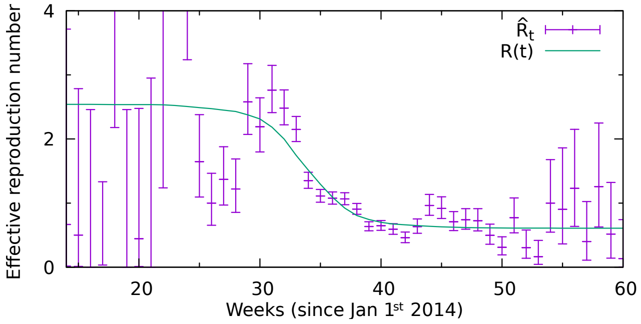

Figure A: Weekly estimates of the effective reproduction number $\hat{R}_t$ in Liberia (purple crosses). Fitted $R(t)$ (green line) with $R_H = 2.0$, $R_L = 0.6$ and $N_H = 2\,500$ (see equation 1).

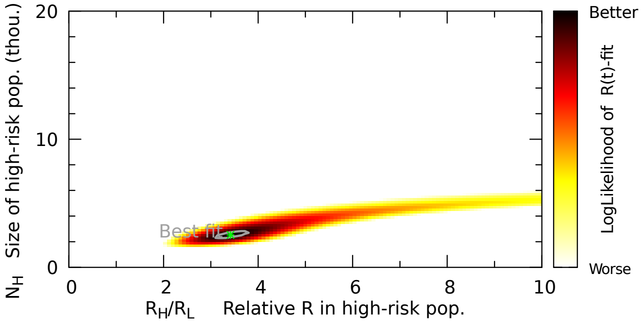

Figure B: Quality of the fit shown in figure 1 for different parameter choices. The grey line marks the 95% confidence interval.

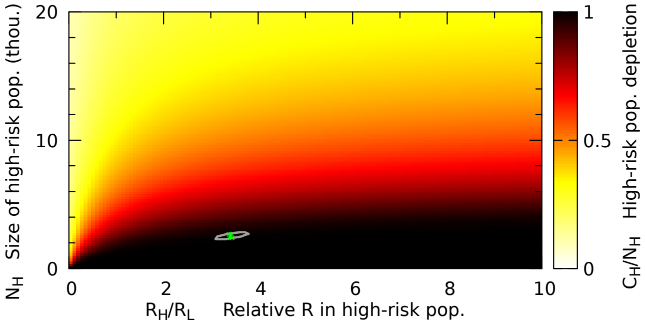

Figure C: Expected final fraction of infected high risk population for different parameter choices. The matching R_0 for is shown in figure 6.

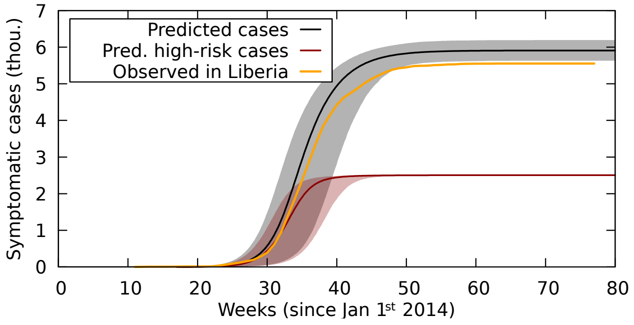

Figure D: Observed case count and results of the agent based simulations using the fitted parameter set (see green star in fig. 2 and fit in fig. 1). Orange line: Observed case count in Liberia. Black line: Mean total case count. Red line: Mean case count in the high risk population. Grey and red areas: Prediction interval (95%) for case counts (95% of our simulations produce case counts within this area).

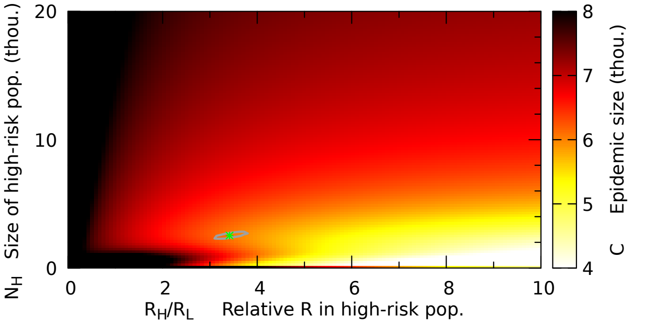

Figure E: Expected total cases for different parameter choices. The matching R_0 for is shown in figure 6.

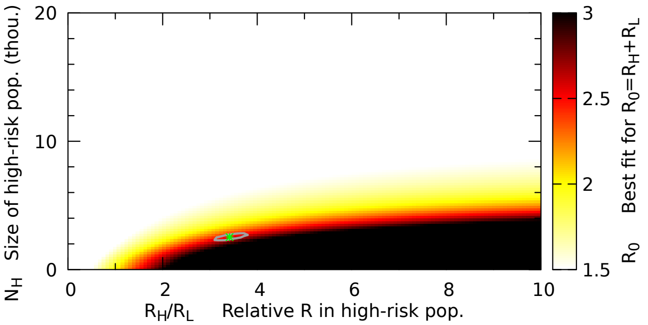

Figure F: Best R_0 for fit shown in figure 1 for different parameter choices.

Sierra Leone

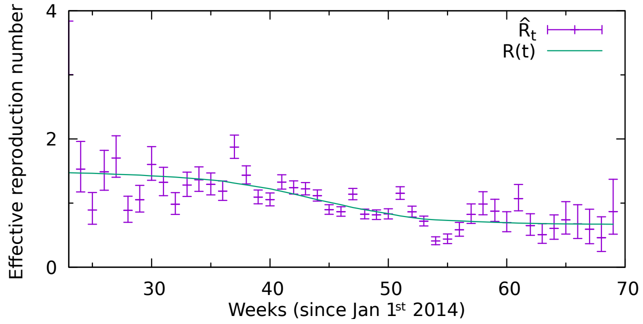

Figure G: Weekly estimates of the effective reproduction number $\hat{R}_t$ and $R_t(C_t)$ (see equation 1).

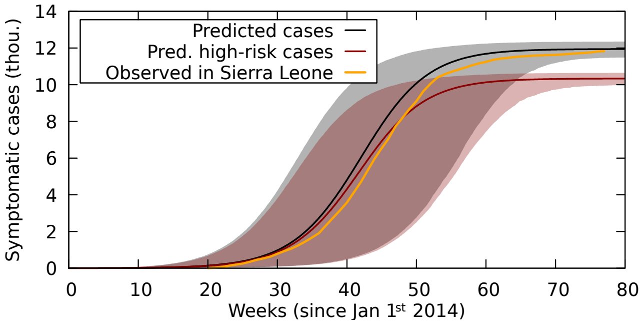

Figure H: Simulated dynamics with agent based stochastic model with same parameters as the fit in figure 1.

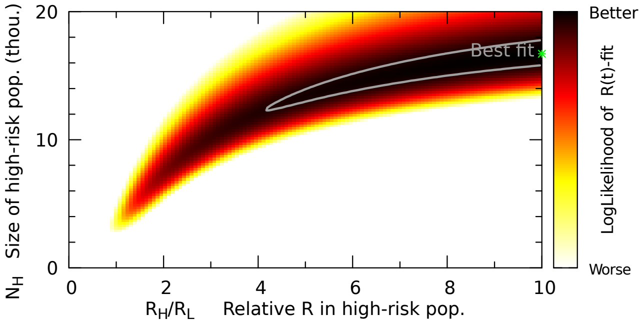

Figure I: Quality of the fit shown in figure 1 for different parameter choices. The matching R_0 for is shown in figure 6.

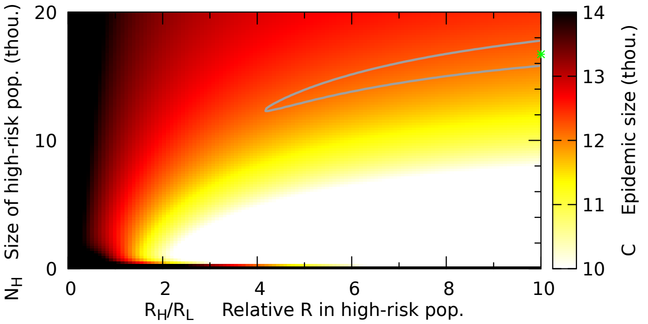

Figure J: Expected total cases for different parameter choices. The matching R_0 for is shown in figure 6.

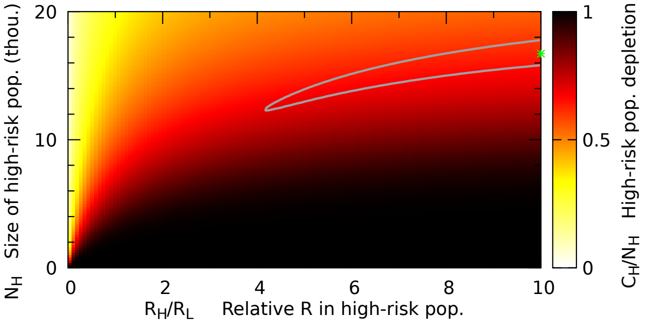

Figure K: Expected final fraction of infected subpopulation for different parameter choices. The matching R_0 for is shown in figure 6.

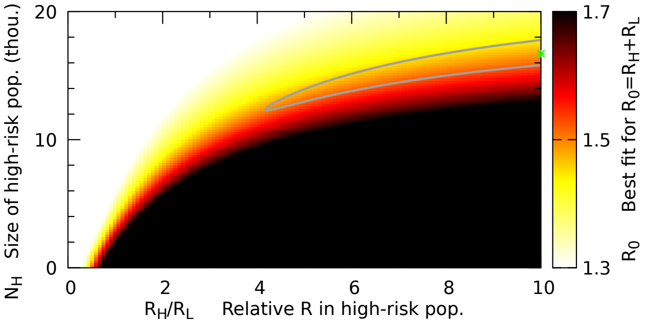

Figure L: Best R_0 for fit shown in figure 7 for different parameter choices.

Guinea

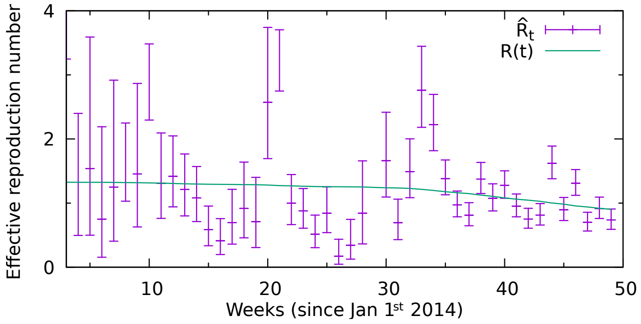

Figure M: Weekly estimates of the effective reproduction number $\hat{R}_t$ and $R_t(C_t)$ (see equation 1).

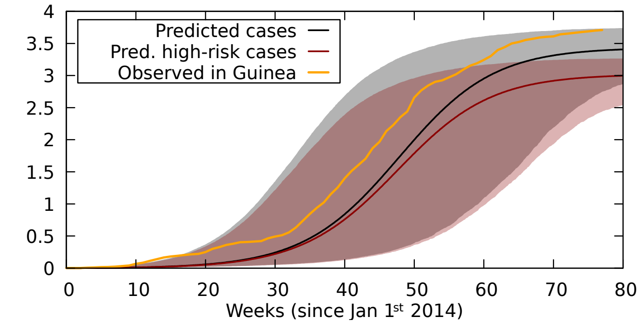

Figure N: Simulated dynamics with agent based stochastic model with same parameters as the fit in figure 13.

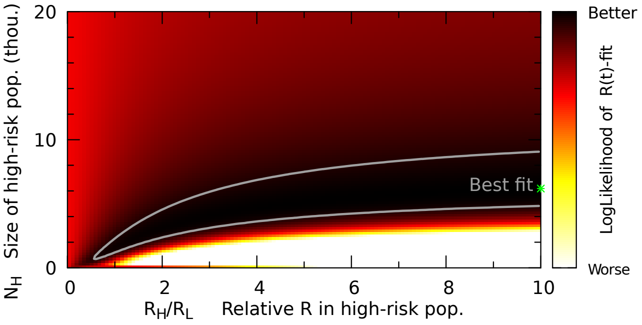

Figure O: Quality of the fit shown in figure 1 for different parameter choices. The matching R_0 for is shown in figure 18.

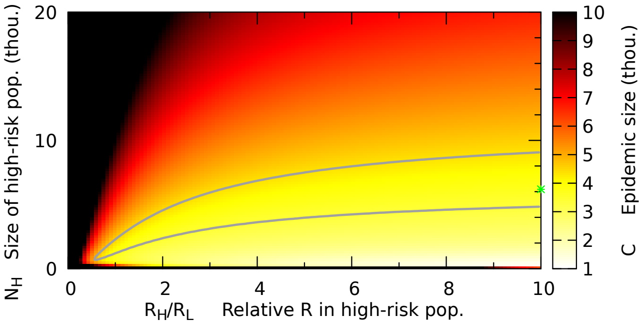

Figure P: Expected total cases for different parameter choices. The matching R_0 for is shown in figure 6.

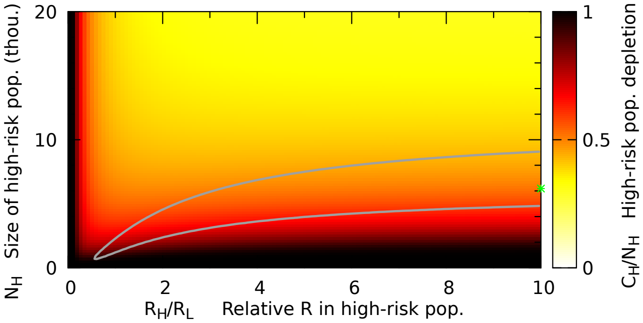

Figure Q: Expected final fraction of infected subpopulation for different parameter choices. The matching R_0 for is shown in figure 18.

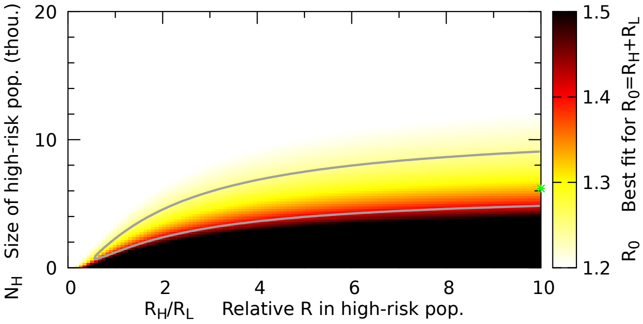

Figure R: Best R_0 for fit shown in figure 13 for different parameter choices.
